# Non-invasive brain neuromodulation techniques for chronic low back pain

**DOI:** 10.3389/fnmol.2022.1032617

**Published:** 2022-10-19

**Authors:** Tian-Tian Chang, Yu-Hao Chang, Shu-Hao Du, Pei-Jie Chen, Xue-Qiang Wang

**Affiliations:** ^1^Department of Sport Rehabilitation, Shanghai University of Sport, Shanghai, China; ^2^Department of Luoyang Postgraduate Training, Henan University of Traditional Chinese Medicine, Luoyang, China; ^3^Department of Rehabilitation Medicine, Shanghai Shangti Orthopaedic Hospital, Shanghai, China; ^4^Shanghai Key Lab of Human Performance, Shanghai University of Sport, Shanghai, China

**Keywords:** pain, mechanisms, brain neuromodulation, rTMS, tDCS, low back pain

## Abstract

Structural and functional changes of the brain occur in many chronic pain conditions, including chronic low back pain (CLBP), and these brain abnormalities can be reversed by effective treatment. Research on the clinical applications of non-invasive brain neuromodulation (NIBS) techniques for chronic pain is increasing. Unfortunately, little is known about the effectiveness of NIBS on CLBP, which limits its application in clinical pain management. Therefore, we summarized the effectiveness and limitations of NIBS techniques on CLBP management and described the effects and mechanisms of NIBS approaches on CLBP in this review. Overall, NIBS may be effective for the treatment of CLBP. And the analgesic mechanisms of NIBS for CLBP may involve the regulation of pain signal pathway, synaptic plasticity, neuroprotective effect, neuroinflammation modulation, and variations in cerebral blood flow and metabolism. Current NIBS studies for CLBP have limitations, such as small sample size, relative low quality of evidence, and lack of mechanistic studies. Further studies on the effect of NIBS are needed, especially randomized controlled trials with high quality and large sample size.

## Introduction

Low back pain, which refers to pain and discomfort in the lumbosacral region, is a highly prevalent condition with high burden worldwide (Balagué et al., 2012; Smith et al., 2022). The point prevalence and lifetime prevalence of low back pain are 7.83% (95% confidence interval [CI]: 7.04–8.64) and 84% (Balagué et al., 2012). Moreover, the prevalence of low back pain and years lived with disability caused by low back pain are expected to increase as a result of population growth and aging (Hartvigsen et al., 2018). In the US, the annual expenditures for low back pain exceed $100 billion (Katz, 2006), and the annual costs for each patient reached $8386 (Gore et al., 2012). Chronic low back pain (CLBP) is defined as low back pain with a course of over 12 weeks or 3 months (Andersson, 1999). Approximately two-thirds of low back pain cases will develop CLBP after the first episode (Morlion, 2013). Although CLBP has been recognized as a crucial global health and socioeconomic problem, its treatment has tremendous potential for improvement (Knezevic et al., 2021).

Alterations exist in the structure and function of several neural networks in patients with CLBP (Kregel et al., 2015; Ng et al., 2018). Brain imaging studies have confirmed the structural changes in gray matter in the thalamus, dorsolateral prefrontal cortex, temporal lobes, insula, and the primary somatosensory cortex in patients with CLBP (Giesecke et al., 2004; Vlaeyen et al., 2018). Moreover, patients with CLBP had increased activities in certain cortical and subcortical regions (such as the prefrontal cortex and cingulate cortex) and reduced activities in pain-relief areas. These neuroanatomical and functional abnormalities in the brain can be reversed by an effective treatment (Seminowicz et al., 2011). Therefore, directly targeting the brain region involved in pain processing may be an effective treatment for CLBP. Non-invasive brain neuromodulation (NIBS) was defined as any brain stimulation technique that directly modulates brain activity and the neural network involved in pain processing but does not require invasive methods (Jenkins and Tepper, 2011; Romanella et al., 2020). Repetitive transcranial magnetic stimulation (rTMS) and transcranial direct current stimulation (tDCS) are the two most commonly used forms of NIBS (O’Connell et al., 2018). Recently, NIBS has been used for the management of chronic pain, including neuropathic pain (Zhang et al., 2021) and chronic headache (Cheng et al., 2022). Many studies also explored the effect of NIBS on patients with CLBP and observed positive results, but the placebo effect should not be ignored (Ambriz-Tututi et al., 2016; Jiang et al., 2020). However, to our best knowledge, only one review summarized the effectiveness of NIBS for chronic non-specific low back pain, and no study summarized its mechanism (Patricio et al., 2021). Therefore, the aim of this review is to outline the effect of NIBS on patients with CLBP and summarize the possible mechanism of action.

## Non-invasive brain neuromodulation for chronic low back pain

### Repetitive transcranial magnetic stimulation for chronic low back pain

Repetitive transcranial magnetic stimulation, an FDA-approved NIBS technique, alters the excitability of the cerebral cortex by generating strong magnetic and electric fields through the stimulation coil applied over the scalp (Klomjai et al., 2015). Generally, rTMS can be divided into high-frequency rTMS (>1 Hz, HF-rTMS) and low-frequency rTMS (≤1 Hz, LF-rTMS) according to their frequencies (Pascual-Leone et al., 1999; Haraldsson et al., 2004; Guse et al., 2010).

Johnson et al. (2006) recruited 17 patients with CLBP and observed a decrease in brief pain ratings after a single session of HF-rTMS stimulation. They also found a remarkable decrease in the temperature for cold pain thresholds and a significant increase in the temperature for heat pain thresholds after a single session of HF-rTMS stimulation (Johnson et al., 2006; Table 1). And pain intensity was remarkably decreased in CLBP patients after repeated HF-rTMS session stimulation. Besides, rTMS had a greater analgesic effect without evident side effects compared with physical therapy (Ambriz-Tututi et al., 2016). Many lines of evidence suggested that the presence of attendant symptoms, such as depression and sleep disturbance, interferes with CLBP treatment and were associated with worse treatment outcomes (Sullivan et al., 1992; Nijs et al., 2018). Park et al. (2014) attempted to explore the effects of rTMS in treating depression and insomnia with CLBP and observed positive results. Therefore, rTMS maybe yield an optimal result in the concurrent treatment of these symptoms in patients suffering from CLBP and depression.

**TABLE 1:** Non-invasive brain neuromodulation (NIBS) studies for chronic low back pain.

Study author, year	Study type	Sample size (E vs. C)	Gender (F/M)	Age	Control	Pain duration (month)	Parameters and dosage	Session schedule	NIBS combined with other interventions	Outcome measure	Follow-up
**rTMS**											
Ambriz-Tututi et al., 2016	RCT	67 (41 vs. 26)	rTMS: 27/14 sham: 18/8	rTMS: 52.57 ± 10.5 sham: 51.5 ± 12.3	Sham	rTMS: 85.2 ± 31.2 sham: 58.8 ± 30	Coil type: F-8 stimulation site: M1 frequency: 20 Hz (10 pulse trains of 10 s with 28 s inter-train interval) intensity: 95%RMT	Five sessions during the first week and 8 more sessions on weeks 3, 4, 6, 8, 12, 20, 28, and 36. At the end of protocol, sham group received real rTMS for a week, rTMS group received sham session.	none	VAS; SF-MPQ; SF-36	none
Park et al., 2014	Case report	2	2/0	Patient 1: 65 Patient 2: 61	NA	Patient 1: 60 Patient 2: 12	Coil type: F-8 Stimulation site: PFC Frequency: 1 Hz (1200 pulses/session) Intensity: 100%RMT	Patient 1: 5 sessions/week; 4 weeks; Patient 2: 5 sessions/week; 3 weeks	None	NRS;BDI; ISI, PDI	None
Johnson et al., 2006	RCT (cross-over)	17 (17 vs. 17)	10/7	Mean age: 43.5 (range: 28–74)	Sham	NR (over 12 m)	Coil type: F-8 stimulation site: M1 Frequency: 20 Hz (12.5 trains with 28 s inter-train interval, total 500 pulses/session) Intensity: 95%RMT	A single session	None	BPI; QST	None
**tDCS**											
McPhee and Graven-Nielsen, 2021	RCT (cross-over)	12 (12 vs. 12)	9/3	All: 28.6 ± 5.9	Sham	All: 63.6 ± 31.2	Mode: Anodal stimulation site: Anodal over medial prefrontal region (Fz), Cathode over the forehead (Fp1, Fp2, F7, and F8) Intensity: 2 mA Session duration: 20 min	Daily stimulation; 3 consecutive days	None	VAS, RMDQ, MPQ, PPT	21 days
Jiang et al., 2020	RCT	51 (26 vs. 25)	27/24	tDCS: 39.9 ± 14.2 sham: 44.1 ± 13.0	Sham	All: 27.5 ± 47.6	Mode: Anodal stimulation site: Anodal over C3/C4 (contralateral of pain), Cathode over the contralateral supraorbital area Intensity: 2 mA session duration: 20 min	A single session	None	NRS	None
Jafarzadeh et al., 2019	RCT	36 (12 vs. 12 vs. 12)	6/30	All: 31.6 ± 4.9	Sham + postural training; postural training	NR (chronic)	Mode: Anodal stimulation site: Anodal over left M1 (C3), Cathode over the right contralateral supraorbital area intensity: 2 mA Session duration: 20 min	3 session/week, 2 weeks	Concomitant with postural training for 20 min, 3 sessions/week, 2 weeks	VAS, BBS	1-month
Mariano et al., 2019	RCT	21 (10 vs. 11)	7/23	All: 63.1 ± 10.5	Sham	NR (chronic)	Mode: Cathodal Stimulation site: Cathodal over dACC (C3), Anodal over the right contralateral mastoid Intensity: 2 mA session duration: 20 min	Daily stimulation; 10 consecutive weekdays	None	DVPRS, RMDQ, PHQ-9,	6 weeks
Straudi et al., 2018	RCT	35 (18 vs. 17)	26/9	All: 55.1 ± 12.5	Sham + group exercise	All: 104.4 ± 92.4	Mode: Anodal stimulation site: Anodal over M1 (contralateral of pain), Cathode over the contralateral supraorbital area Intensity: 2 mA Session duration: 20 min	Daily stimulation; 5 consecutive days	After 5 sessions tDCS, 1 h neurophysiology lesson; and 10 h muscle stabilization and mobilization exercise (1 h/session; 2–3 sessions/week, 4 weeks, total 10 sessions)	VAS, RMDQ, EQ-5D, PHQ-9	1-month
Hazime et al., 2017	RCT	92 (23 vs. 23 vs. 23 vs. 23)	69/23	Real tDCS + sham PES: 51.9 ± 9.9; sham tDCS + real PES: 53.0 ± 9.9; real tDCS + real PES: 51.3 ± 9.9; sham tDCS + sham PES: 54.1 ± 9.8	Sham	Real tDCS + sham PES: 91.6 ± 108.3; sham tDCS + real PES: 59.7 ± 59.7; real tDCS + real PES: 37.3 ± 39.4; sham tDCS + sham PES: 69.2 ± 92.7	Mode: Anodal stimulation site: Anodal over C3 or C4 (contralateral of pain), Cathode over the contralateral supraorbital region Intensity: 2 mA Session duration: 20 min	3 sessions/week; 4 weeks	Concomitant with real or sham PES (100 Hz, 40 min); PES electrodes placed over lumbar segment (most painful site); 3 sessions/week; 4 week	NRS, RMDQ	12, 24 weeks
Luedtke et al., 2015	RCT	135 (67 vs. 68)	63/72	tDCS: 45 ± 9 Sham: 44 ± 10	Sham	tDCS: 23 ± 49 Sham: 19 ± 29	Mode: Anodal stimulation site: Anodal over M1 (contralateral of pain), Cathode over the contralateral supraorbital region Intensity: 2 mA Session duration: 20 min	Daily stimulation; 5 consecutive days	After 5 sessions tDCS stimulation, the cognitive-behavioral management program was performed (5 h/day, 4 weeks, total 80 h)	VAS, ODI	None
Schabrun et al., 2014	RCT (cross- over)	16 (16 vs. 16 vs. 16 vs. 16)	7/9	All: 30 ± 2	Sham	All: 50.4 ± 8.4	Mode: Anodal Stimulation site: Anodal over M1 (contralateral of pain), Cathode over the contralateral supraorbital region Intensity: 1 mA Session duration: 30 min	A single session	Concomitant with real or sham PES (2 Hz, 30 min); PES electrodes placed over lumbar segment, a single session	NRS, PPT, TPD	3 days
O’Connell et al., 2013	RCT (cross-over)	8 (8 vs. 8)	7/1	All: 45 ± 10	Sham	NR (over 12m)	Mode: Anodal Stimulation site: Anodal over M1, Cathode over the contralateral supraorbital region Intensity: 2 mA session duration: 20 min	5 sessions/week, 3 weeks	None	VAS, RMDQ, cognitive function	3 weeks
Luedtke et al., 2012a	RCT (cross- over)	15 (15 vs. 15 vs. 15)	9/6	Mean age: 48.7 (range: 30–70)	Anodal vs. cathodal vs. sham	All: 134.4 m (mean value)	Mode: Anodal or Cathodal Stimulation site: Anodal over left M1, Cathode over the right supraorbital region Intensity: 1 mA Session duration: 15 min	A single session	None	QST	None
**tACS**											
Ahn et al., 2019	RCT (cross-over)	20 (20 vs. 20)	12/8	NS	Sham	84.8 ± 70	Stimulation site: 2 electrode on F3 and F4 and 1 at Pz Frequency: 10 Hz Intensity: 1 mA Session duration: 40 min	A single session	None	DVPRS, ODI	None

E, experimental group; C, control group; M1, primary motor cortex; PFC, prefrontal cortex; RMT, resting motor threshold; F-8, figure-of-8 coil; VAS, visual analog scale; NRS, numerical rating scale; SF-MPQ, Short Form McGill pain questionnaire; SF-36, Short Form 36 Health Survey; BDI, Beck Depression Inventory; ISI, Insomnia Severity Index; PDI, the Pain Disability Index; QST, quantitative sensory testing; BPI, Brief Pain Inventory; PPT, pressure pain thresholds; RMDQ, Roland Morris Disability Questionnaire; ODI, Oswestry disability index; DVPRS, defense and veterans pain rating scale; BBS, Berg Balance Scale; MPQ, McGill Pain Questionnaire; TPD, two-point discrimination; PHQ-9, The Patient Health Questionnaire-9; EQ-5D, EuroQuol-5 Dimension; Hz, hertz; NR, not reported; NA, not applicable.

These investigations provided preliminary confirmation of the therapeutic effects of rTMS on CLBP. According to the updated guidelines on the therapeutic use of rTMS, level A (definite efficacy) evidence strongly suggested that rTMS is effective for managing neuropathic pain (Lefaucheur et al., 2020; Leung et al., 2020). However, more high-quality and large-scale randomized controlled trial (RCT) studies are needed to further support the benefit of rTMS for CLBP. Besides, no study has compared the effects of different NIBS techniques for CLBP. Whether the use of rTMS may offer more advantages than other NIBS techniques is unknown.

### Transcranial direct current stimulation for chronic low back pain

Transcranial direct current stimulation modulates cortical excitability by passing positively or negatively charged currents (a weak current, 0.5–2 mA) using at least two surface electrodes on the scalp (Luedtke et al., 2012b; Chase et al., 2020). In many countries, tDCS can be used as an off-label treatment, and its official regulatory status is under development (Fregni et al., 2015). In the United States, no clinical indications have been approved for the use of tDCS, but tDCS is widely studied for the treatment of chronic pain, and CLBP is not an exception (Pacheco-Barrios et al., 2020).

Some studies used a single session to explore the therapeutic effect of tDCS on CLBP. A single, 20-min session of anodal tDCS stimulation at 2 mA targeting M1 remarkably improved the pain in the tDCS group compared with the sham group (Straudi et al., 2018; Jiang et al., 2020). Schabrun et al. (2014) observed that the analgesic effect of tDCS (a single session, 30 min, 1 mA) was maintained for at least 3 days. But Luedtke et al. (2012a) used a single, 15-min session of M1-tDCS stimulation in CLBP patients, they found that tDCS does not dramatically change the experimentally induced pain. Although they choose the stimulation paradigm most commonly used in most studies on experimentally induced pain in healthy subjects, this stimulation intensity and duration may not be sufficient for patients with chronic pain (Fregni et al., 2006; Soler et al., 2010). Only one meta-analysis summarized the effect of NIBS on CLBP and also found that a single session of NIBS remarkably reduced the pain intensity in patients with CLBP (Patricio et al., 2021). Therefore, these pieces of evidence showed that a single session of tDCS seem to be effective in CBLP treatment.

The effects of multiple sessions of tDCS on CLBP have been investigated, but the immediate changes after intervention are not remarkable (Alwardat et al., 2020; McPhee and Graven-Nielsen, 2021). The therapeutic effect of rTMS could be enhanced by multiple sessions of stimulation (Quesada et al., 2018). Similarly, the effect of tDCS may accumulate over time but may take a longer period to fully manifest (Bikson et al., 2018; Sampaio-Junior et al., 2018). Although Mariano et al. (2019) did not observe an immediate reduction in pain in the tDCS group after 10 sessions of tDCS, there was a remarkable decrease in disability, pain interference, and depression symptoms at 6 weeks of follow-up. Besides, the cognitive function of patients with CLBP appears to have a promotion at 3 days of follow-up after multiple tDCS intervention (O’Connell et al., 2013). This delayed effect also appears in the treatment of depression using rTMS or electroconvulsive therapy. In addition to the delayed effect, the negative results were associated with the less rigid inclusion criteria because of the complexity and heterogeneity of CLBP (Luedtke et al., 2015).

In addition to tDCS as monotherapy, some studies also explored the combined effect of tDCS and other interventions in the treatment of CLBP (Luedtke et al., 2015; Hazime et al., 2017). An increased treatment effect in postural stability, balance, and pain was found by adding tDCS to postural training in patients with CLBP (Jafarzadeh et al., 2019). tDCS remarkably improved the therapeutic effect of group exercises on pain and psychological well-being (particularly depression) in patients with CLBP (Straudi et al., 2018). In addition, a single session of combined tDCS and peripheral electrical stimulation (PES) could significantly increase the pressure pain thresholds and pain-free range of lumbar flexion and decrease the two-point discrimination threshold in CLBP patients compared with PES alone, and the effect was maintained for at least 3 days (Schabrun et al., 2014). Recently, Fregni et al. (2021) proposed that anodal tDCS should be moderately recommended (level B) in reducing chronic pain, such as neuropathic pain, fibromyalgia pain, and migraine pain. But for CLBP, the analgesic effect of tDCS appears to be inadequate. Given the pain relief after a single session of tDCS and the additional effect of tDCS in combination with other therapies on CLBP, the analgesic effect of tDCS for CLBP cannot be entirely denied based on the limited studies (Schabrun et al., 2014; Straudi et al., 2018; Jiang et al., 2020).

### Transcranial alternating current stimulation for chronic low back pain

Transcranial alternating current stimulation (tACS) can regulate neural oscillation by applying an alternating current with a sinusoidal pattern to the scalp (Woods et al., 2016). Although it shares basic electrode montage and low-intensity features with tDCS, the functional interpretations of the two electrodes are different (Elyamany et al., 2021). Anodal and cathodal tDCS provide positive and negative currents, respectively. One electrode is an anode, and the other one is a cathode during the half cycle of the tACS oscillation cycle, and this pattern is reversed during the other half of the cycle (Antal and Herrmann, 2016). The use of tACS has a strong theoretical foundation and context for the treatment of chronic pain (Tan et al., 2019; Zhou et al., 2019a). The neural oscillation signals in the alpha and gamma bands during the pre-stimuli period negatively regulate the perception of nociceptive stimuli (Tu et al., 2016). Previous studies suggested a remarkable reduction in the experimental pain induced by pressure pain stimulator in healthy subjects after tACS at alpha frequency (Arendsen et al., 2018). tACS regulates pain intensity by altering neural oscillations (especially in alpha and gamma neural oscillation signals) in patients with chronic pain (Helfrich et al., 2014; Vossen et al., 2015). Although it is appealing, the application of tACS seems to be rare in pain management. Only one study has explored the analgesic effect of tACS on CLBP. Ahn et al. (2019) recruited 20 patients with CLBP and performed a crossover RCT. They observed a substantial pain reduction after a single session of 10 Hz tACS stimulation over the F3 and F4 (10–20 international coordinate system) for 40 min. Moreover, tACS stimulation induced an increase in the intensity of the alpha oscillation signal in the somatosensory area, which was closely associated with pain relief in patients with CLBP (Ahn et al., 2019).

## Mechanisms of non-invasive brain neuromodulation for chronic low back pain

### Regulation of pain-related signal pathway

Patients with CLBP exhibit changes in brain networks, including pain modulation network, attention network, and default mode network (Kregel et al., 2015; Ng et al., 2018; Vlaeyen et al., 2018). There was a significant difference in functional connectivity in the periaqueductal gray–centered pain modulation network between the patients with CLBP and healthy controls (Yu et al., 2014). This was compatible with the impairments of the descending pain modulation reported in patients with CLBP (Hemington and Coulombe, 2015; Yu et al., 2020). These pieces of evidence indicated the dysfunction in the pain modulatory system in patients with CLBP. Many studies have shown that rTMS and tDCS can modulate neural activity in brain structures associated with pain processing.

HF-rTMS increases cortical excitability in the stimulation site, whereas LF-rTMS stimulation decreases cortical excitability. rTMS can produce a local effect by stimulating neurons directly below the coil and induce distant effects through structural white matter connectivity (Valero-Cabré et al., 2017). Many studies have shown that rTMS can regulate neural activity in certain cortical and subcortical areas (e.g., thalamic and various remote structures) to achieve analgesic effects. In particular, rTMS directly stimulates the thalamus through the corticothalamic projection system to inhibit the transmission of injury information through the spinal thalamic pathway (Bestmann et al., 2004). And the bilateral analgesic effects induced by unilateral rTMS stimulation may be due to the activation of certain structures (e.g., periaqueductal gray) involved in the descending inhibitory controls (Pagano et al., 2012; Moisset et al., 2015).

**FIGURE 1:**
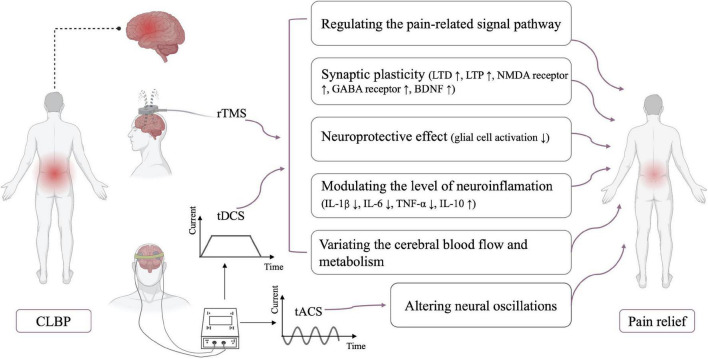
Neurophysiological mechanisms of NIBS. CLBP, chronic low back pain; LTD, long-term depression; LTP, long-term potentiation; NMDA receptor, *N*-methyl-D-aspartate receptor; GABA receptor, gamma-aminobutyric acid receptor; BDNF, brain-derived neurotrophic factor; IL, interleukin; TNF, tumor necrosis factor.

Cathodal tDCS decreases cortical excitability, whereas anodal tDCS increases cortical excitability; the net effect depends on variations in the overall network balance (Truong and Bikson, 2018). In addition to the modulation of the activity in the stimulated region, tDCS can induce changes in structural and functional connections in unstimulated brain regions (Cummiford et al., 2016; Lin et al., 2017). A single session of M1-anodal tDCS can activate the activity of the left medial prefrontal cortex, right caudate, and pontine nuclei and inhibit the activity of the left precentral gyrus (Meeker et al., 2019). Moreover, pain reduction was related to the decreased functional connectivities of the ventral lateral thalamus to the posterior insula, primary motor cortex, and primary somatosensory cortices (Cummiford et al., 2016). These studies indicated that rTMS and tDCS can induce pain relief by regulating the activity of the primary nociceptive processing and inhibitory regions of the brain (Figure 1).

### Synaptic plasticity

The subsequent effects of rTMS and tDCS beyond the stimulation period have been considered as the result of long-term synaptic plasticity (Hosomi et al., 2013; Agboada et al., 2020; Sudbrack-Oliveira et al., 2021). The mechanisms of synaptic plasticity are very complex, and many factors can induce long-term synaptic plasticity, especially the long-term potentiation (LTP) and long-term depression (LTD) phenomena (Connor and Wang, 2016). Similar to basic synaptic physiology, LTP enhances synaptic strength, whereas LTD results in the reduction of synaptic strength (Duffau, 2006). LTP is usually caused by high-frequency rTMS, whereas LTD is induced by low-frequency rTMS (Stanton and Sejnowski, 1989; Artola and Singer, 1993). rTMS and tDCS interact with a variety of neurotransmitters, such as the glutamatergic and GABAergic agents. *N*-methyl-D-aspartate (NMDA) receptor is among the major molecular channels controlling synaptic plasticity, and the after-effects of rTMS and tDCS are dependent on the NMDA receptor (Stagg and Nitsche, 2011; Muller et al., 2014). Furthermore, the analgesic effect of rTMS is attenuated by the use of glutamate antagonists (such as ketamine) prior to rTMS stimulation (Ciampi de Andrade et al., 2014). rTMS enhanced and reduced motor cortex excitability after using type A receptors for gamma-aminobutyric acid (GABA) receptor agonist and antagonist, respectively (Hsieh et al., 2012). The enhanced and prolonged effects of tDCS are also observed after the administration of GABA receptor agonist, lorazepam (Nitsche et al., 2004). rTMS/tDCS regulates NMDA expression and GABA release to induce LTP or LTD, leading to synaptic plasticity. In addition, brain-derived neurotrophic factor (BDNF) is believed to be an important driving force behind synaptic plasticity (Kowiański et al., 2018; Zhou et al., 2019b). Animal studies also showed that rTMS and tDCS could modulate BDNF expression to enhance synaptic plasticity (da Silva Moreira et al., 2016; Shang et al., 2016).

### Neuroprotective effect

Glial activation is present in various chronic diseases, including CLBP (Grace, 2019; Albrecht et al., 2021). Loggia et al. (2015) found an increased level of glial cell activation marker (translocator protein) in the brains of patients with CLBP. Some studies confirmed that glial cell activation is the key factor in the development of chronic pain (Grace, 2019; Torrado-Carvajal et al., 2021). Activated glial cells (such as microglia and astrocytes) can produce various toxic substances, such as cytokines and nitric oxide, thereby aggravating apoptosis (Watkins et al., 2007). Suppression of microglia and astrocyte activation can reverse or reduce chronic pain (Raghavendra et al., 2003; Zheng et al., 2021). Some studies suggested that rTMS may reduce pain by inhibiting the activity and proliferation of microglia and astrocyte in the L4–L6 dorsal and ventral horns of the spinal cord (Kim et al., 2013; Yang et al., 2018). Besides, the activation level of astrocytes in the cerebrospinal fluid was significantly decreased after a single session of tDCS stimulation (Callai et al., 2022). In addition, tDCS and rTMS improved the B-cell lymphoma-2/Bcl2-associated X ratio and decreased apoptosis (Yoon et al., 2011; Zhang et al., 2020). The above studies suggested that rTMS and tDCS may induce a neuroprotective effect.

### Modulation of neuroinflammation

Mounting evidence suggests that neuroinflammation is associated with CLBP (Torrado-Carvajal et al., 2021; Alshelh et al., 2022). Variations in neuroinflammation level were found in tDCS studies (Cioato et al., 2016; Zhang et al., 2020; Callai et al., 2022). Zhang et al. (2020) found a remarkable reduction in the levels of pro-inflammatory cytokines, such as IL-1β, IL-6, and TNF-α, and a substantial increase in the level of anti-inflammatory cytokines, such as IL-10, after tDCS stimulation. Animal studies also revealed an increase in IL-10 in the prefrontal cortex after rTMS stimulation. This variation in neuroinflammation level may be related to the partial reversal of mechanical ectopic pain and hyperalgesia by rTMS (Toledo et al., 2021). These pieces of evidence may be suggested that NIBS can modulate neuroinflammation.

### Variations in cerebral blood flow and metabolism

Some studies reported that the analgesic effects of rTMS and tDCS may be correlated to variations in regional cerebral blood flow and metabolism. After M1-rTMS stimulation, cerebral blood flow was remarkably increased in the right anterior cingulate cortex and contralateral premotor area but substantially decreased in the right medial prefrontal cortex. Pain reduction was considerably associated with variations in the cerebral blood flow in the anterior cingulate cortex (Tamura et al., 2004). Similarly, the tDCS also induced changes in regional cerebral blood flow (Zheng et al., 2011). Additionally, compared with sham stimulation, active tDCS caused an increase in metabolism in the subgenual anterior cingulate cortex, insula, and medulla and a reduction in metabolism in the left dorsolateral prefrontal cortex in patients with chronic pain (Yoon et al., 2014).

## Conclusion and future directions

This review described the effect of NIBS in patients with CLBP and discussed its possible mechanism of action. Overall, NIBS may be effective for CLBP management, and further studies on the effect of NIBS are needed, particularly RCTs with high quality and large sample size. Relatively few studies have examined the analgesic effect of rTMS on patients with CLBP and found promising results. The analgesic effect of tDCS appears to be suboptimal, but the effect of tDCS on CLBP cannot be entirely denied based on the limited studies. No study has compared the efficacy of different NIBS techniques in patients with CLBP. Further studies are warranted to fill this gap, and more different stimulus paradigms are needed to explore the optimal parameters of tDCS and rTMS. In addition, although the analgesic mechanism of NIBS is not understood, the mechanism of NIBS may involve the regulation of pain signal pathway, synaptic plasticity, neuroprotective effect, neuroinflammation modulation, and variations in cerebral blood flow and metabolism. The mechanisms of NIBS for CLBP can be investigated in the future by combining NIBS with imaging or electrophysiology.

## Author contributions

T-TC and Y-HC drafted the manuscript and searched the literature to identify eligible trials. T-TC and S-HD extracted and analyzed the data. X-QW and P-JC conceived and revised this review. X-QW received the funding for this study. All authors read and approved the final manuscript.
